# FOTCA: hybrid transformer-CNN architecture using AFNO for accurate plant leaf disease image recognition

**DOI:** 10.3389/fpls.2023.1231903

**Published:** 2023-09-12

**Authors:** Bo Hu, Wenqian Jiang, Juan Zeng, Chen Cheng, Laichang He

**Affiliations:** ^1^ School of Information Engineering, Nanchang University, Nanchang, China; ^2^ Department of Radiology, the First Affiliated Hospital of Nanchang University, Nanchang, China; ^3^ Second Clinical Medical College, Nanchang University, Nanchang, China; ^4^ School of Mathematics and Statistics, Huazhong University of Science and Technology, Wuhan, China

**Keywords:** plant leaf disease image recognition, hybrid architecture, transformer-based models, adaptive Fourier Neural Operator, deep learning

## Abstract

Plants are widely grown around the world and have high economic benefits. plant leaf diseases not only negatively affect the healthy growth and development of plants, but also have a negative impact on the environment. While traditional manual methods of identifying plant pests and diseases are costly, inefficient and inaccurate, computer vision technologies can avoid these drawbacks and also achieve shorter control times and associated cost reductions. The focusing mechanism of Transformer-based models(such as Visual Transformer) improves image interpretability and enhances the achievements of convolutional neural network (CNN) in image recognition, but Visual Transformer(ViT) performs poorly on small and medium-sized datasets. Therefore, in this paper, we propose a new hybrid architecture named FOTCA, which uses Transformer architecture based on adaptive Fourier Neural Operators(AFNO) to extract the global features in advance, and further down sampling by convolutional kernel to extract local features in a hybrid manner. To avoid the poor performance of Transformer-based architecture on small datasets, we adopt the idea of migration learning to make the model have good scientific generalization on OOD (Out-of-Distribution) samples to improve the model’s overall understanding of images. In further experiments, Focal loss and hybrid architecture can greatly improve the convergence speed and recognition accuracy of the model in ablation experiments compared with traditional models. The model proposed in this paper has the best performance with an average recognition accuracy of 99.8% and an F1-score of 0.9931. It is sufficient for deployment in plant leaf disease image recognition.

## 1 Introduction

As one of the main sources of economic growth in many developing countries, it is necessary to produce agricultural crops in a stable and efficient manner (Iqbal et al., 2018). However, the expansion of crops, combined with the overuse of pesticides and the exacerbation of global climate change, has led to an increase in the occurrence and spread of agricultural pests and diseases. Therefore, controlling these pests and diseases is becoming increasingly challenging. Early detection and treatment of such pests and diseases have unique advantages (Sanju and VelammaL, 2021). In the early stages of pest infestation, it is difficult to distinguish the leaves of affected plants from those of normal because of the high interclass variation in colour and profile and the low intraclass variation.

Traditional methods for identifying agricultural pests typically involve visual inspection of crops by farmers and agricultural experts. However, these approaches are costly, time-consuming, highly subjective, and non-transferable (Ouppaphan, 2017). Although some success has been achieved in classifying agricultural images using traditional image processing techniques, these methods face several challenges. First, they often require manual feature extraction, increasing workload. Second, the manually extracted features may not adequately represent the characteristics of agricultural images, leading to semantic gaps. Lastly, the variability and complexity of agricultural images, combined with factors such as image quality and shooting angle, can significantly affect the final recognition results, rendering these techniques unsuitable for large-scale applications (Bisen, 2021; Pan et al., 2022).

In light of relevant research on plant leaf disease identification and fine-grained recognition, we investigated issues associated with the relatively coarse recognition of algorithms in current crop pest identification approaches and their inadequate performance on datasets containing multiple, similar pests and diseases. We recognized the potential of the Transformer-based model and applied it to this domain. However, using the original patch and position embedding(Pap Embedding) would impose limitations on the experiment outcomes. Concurrently, utilizing the adaptive Fourier basis function to convert images to the frequency domain and deploying a CNN-Transformer architecture to separately extract local and global features could enhance the training upper limit. As a solution, we propose a novel hybrid architecture for plant leaf disease image recognition, termed FOTCA (where F and O signify Adaptive Fourier Neural Operator (AFNO) (Guibas et al., 2021) and TCA represents the Transformer-CNN Architecture). This approach addresses and optimizes the convergence issue and generalization capability of the ViT model, further improving training outcomes. Additionally, we evaluate the performance of FOTCA using the Plant-village dataset, a plant leaf disease dataset, to assess its scope and effectiveness.

In summary, this work makes the following three main contributions.

- This article applies an operator called Adaptive Fourier Neural Operator (AFNO) and learnable Fourier features which can replace traditional position encoding. Compared to traditional self-attention, AFNO maps images to frequency domain for better performance.
- A model architecture that integrates both global and local features has been proposed, connecting CNNs and Transformers through inter-level concatenation to achieve coupling of global and local receptive fields. This hybrid architecture, which blends global and local features, can better utilize the extracted features and improve the performance and robustness of the model.
- This article proposes that using Focal Loss as the loss function can effectively enhance the model’s ability to train on difficult samples.

The rest of this article is organized as follows. The Section 3 mainly elucidates the details of the dataset composition and model composition structure, in Section 4 we specify the optimization scheme for settings other than the model.

## 2 Related works

### 2.1 Convolutional Neural Networks

Convolutional Neural Networks (CNNs) have become a popular and powerful tool for image recognition in various fields. In 1998, Lécun et al. (1998) pioneering introduction of the LeNet-5 model laid the fundamental framework for CNNs. In 2012, the AlexNet model (Krizhevsky et al., 2012) used more convolutional layers and a larger parameter space to fit large-scale datasets in ILSVRC (ImageNet Large-Scale Visual Recognition Challenge). It was a groundbreaking demonstration of the advantages of deep neural networks over shallow neural networks, achieving significantly higher accuracy than the runner-up in recognition accuracy. This breakthrough established the status of CNNs in computer vision and brought new opportunities for plant leaf disease identification.

### 2.2 Fine-grained image recognition

State-of-the-art deep learning algorithms for image recognition predominantly rely on publicly available datasets such as ImageNet. Although utilizing these datasets enhances a model’s capability to identify objects in images, including contour features (Wang D. et al., 2021; Zhang and Wang, 2016), discerning different types of pests affecting the same plant leaves in plant leaf disease images remains challenging due to their similar contour features (Kong et al., 2021). Merely transferring a model trained on other data categories to the study of plant leaf disease might not yield the anticipated accuracy. Consequently, models should be encouraged to learn fine-grained features of objects for improved results (Berg et al., 2014; Yang et al., 2018; Chen et al., 2019)

To tackle this issue, numerous researchers have explored convolutional neural networks in fine-grained recognition. For instance, Zhang et al. (2014) developed a model that surmounts these constraints by utilizing deep convolutional features derived from bottom-up region proposals for fine-grained recognition. Multi-proposal Net (Zhang et al., 2016) obtains image blocks through the Edge Box Crop method and incorporates an output layer of key points and visual features to further reinforce the local feature positional relationship between local features and overall information. Deep LAC (Lin et al., 2015) performs part localization, alignment and classification in the same network, and the VLF (valve linkage function) function is proposed for back propagation in Deep LAC, which is able to adaptively reduce the classification and alignment errors and update the localization results.

### 2.3 Transformer-based models for fine-grained visual recognition

As a decoding and encoding architecture model based on attention mechanism, Transformer (Vaswani et al., 2017) has been widely applied in the field of natural language processing. Inspired by this significant achievement, many scholars have transferred the Transformer structure to computer vision tasks and have achieved considerable success (Carion et al., 2020; Dosovitskiy et al., 2020; Wang Y. et al., 2021). The attention mechanism primarily identifies and classifies objects through different parts of an object, thus making it possible to focus on key features of the subject for datasets of plant leaf diseases with small differences between inter-class. As a result, the Transformer structure completes more successful recognition compared to CNN structures. Focusing on this area of study, Cai et al. (2021) introduced a ViT with adaptive attention that adds attention weakening and strengthening modules. This improves the performance of key features while capturing more feature information. Fu et al. (2017) used multiple scales of recurrent attention links to learn the target feature region and used within-scale classification loss and between-scale ranking loss to make the model more focused on the finer-grained features of the target object. He et al. (2022) proposed the TranFG framework, which integrates all original attention weights of the Transformer into one attention map, enabling the model to recognize image blocks and calculate their relationships while utilizing contrastive loss to expand the distance between confusion class feature representations. Sun (2019) improved the loss function in order to enable the classification model to learn features with greater distinction between more difficult to distinguish classes, and used feature map inhibition methods to enable the model to learn subtle differences.

## 3 Materials and methods

### 3.1 Dataset and data pre-processing

In this study, the base dataset was selected from the publicly available plant-village dataset on the web. This is a dataset specifically designed to study the work of various plant leaf disease recognition models, with a total of 54,303 images containing a total of 38 different species of 13 plant species (including apple, blueberry, cherry, corn, grape, orange, bell pepper, potato, raspberry, soybean, pumpkin, strawberry, tomato). Plant images, including non-plant leaf species, were inserted to train the model to recognize non-plant leaf images. The ratio of the training and validation sets was 8:2.

**Figure 1:**
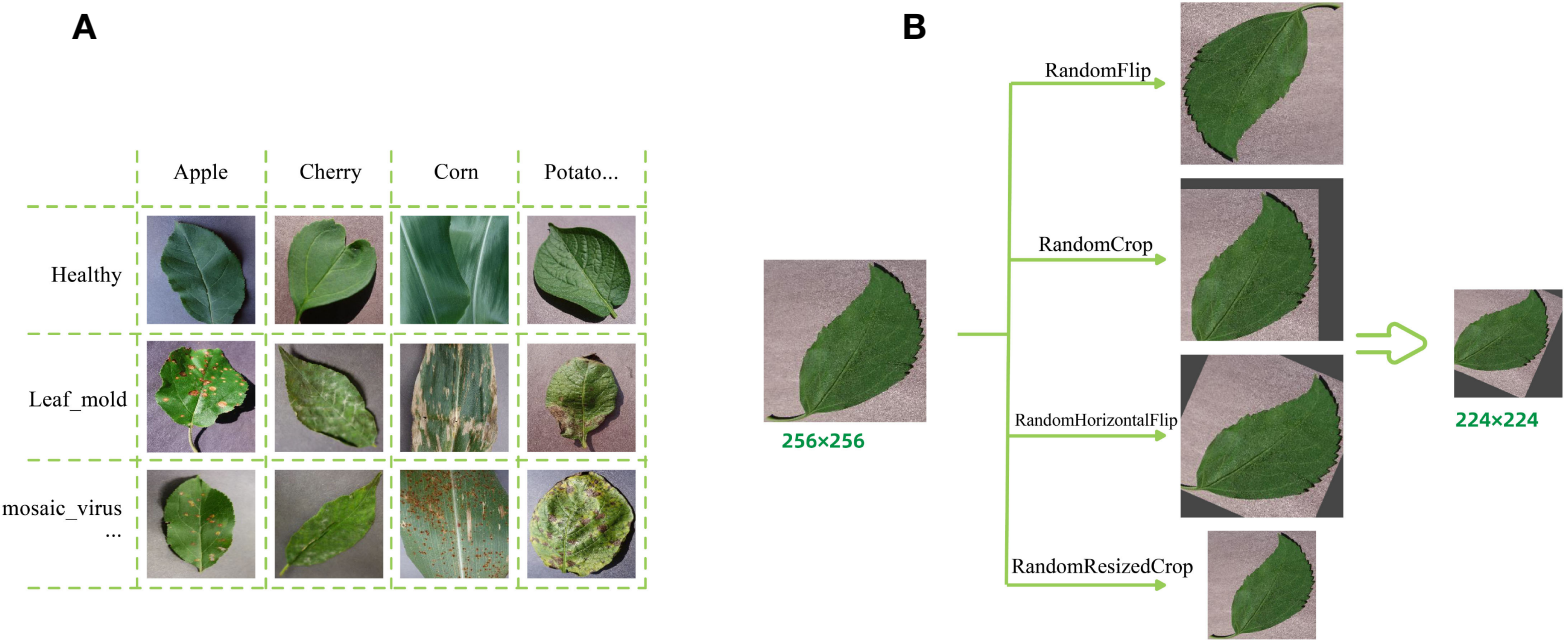
Plant Village dataset presentation and data augmentation examples. **(A)** A partial sample images of the dataset (containing healthy and sick). **(B)** Data augmentation operation examples.

Before inputting the images into the model, the images need to be quantified uniformly. In the first step, the training set is expanded by applying some image enhancement techniques to each image independently superimposed, and the main data enhancement methods used in this study are RandomFlip, RandomCrop, RandomHorizontalFlip and RandomResizeCrop, mainly to eliminate the shift of the final recognition result by the change of the shooting angle in real life, and to prevent the neural network from overfitting phenomenon. In addition, the image size is uniformly adjusted to a square of size 224*224 pixels to facilitate further processing by the model. **Figure 1** shows a portion of the dataset images and the pre-processing process of the images.

### 3.2 Model

**Figure 2:**
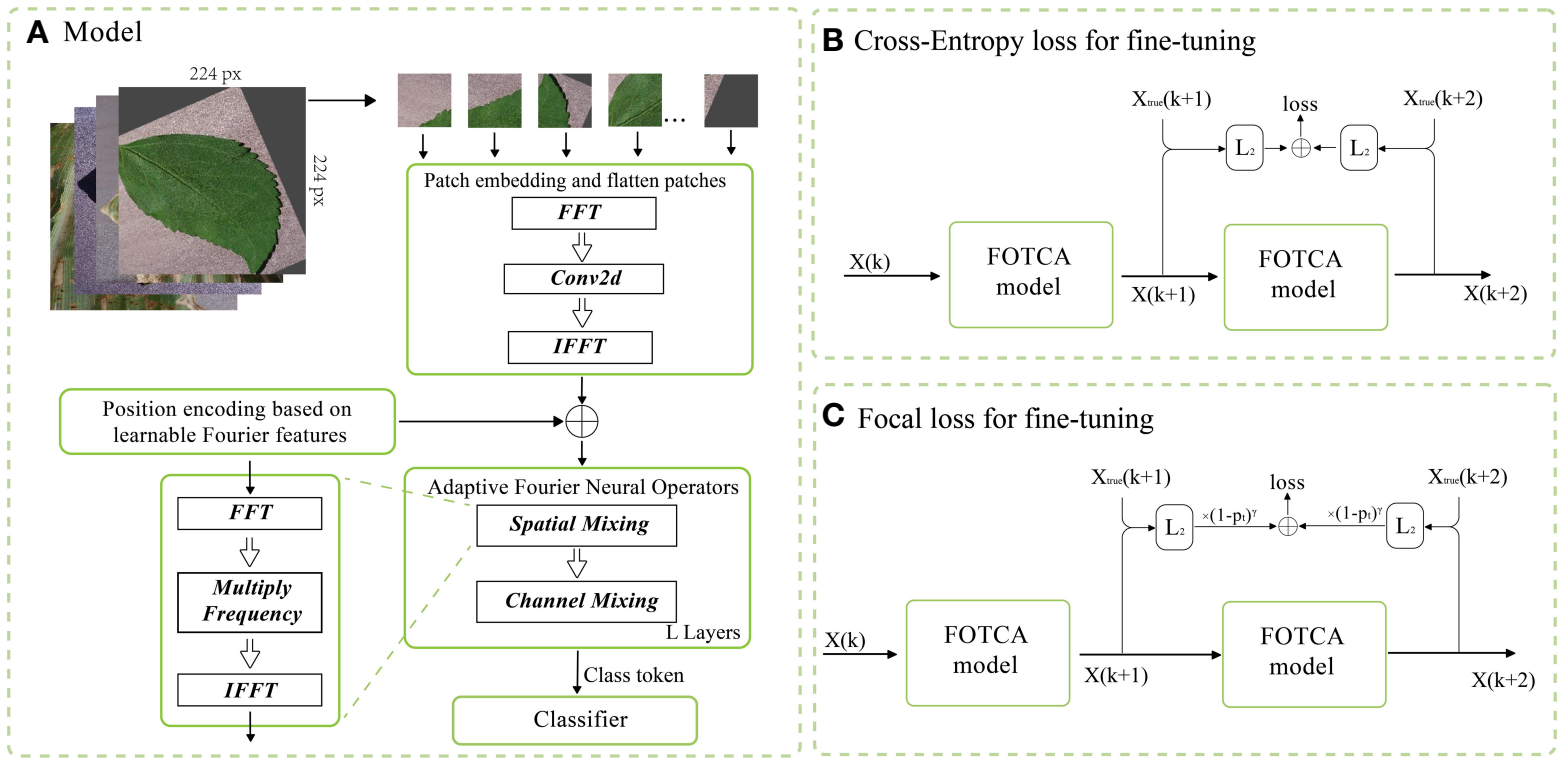
FOTCA model architecture and two loss functions for fine-tuning **(A)** The figure provides an overview of the detailed structure of the entire FOTCA model’s neural network. **(B)** Cross-Entropy loss for fine-tuning. **(C)** Focal loss for fine-tuning.belflow chart.

In this paper, we study applying deep transfer learning to all models in the model and comparison experiments. The Pre-train and Fine-tuning approach in deep transfer learning is the most convenient and reliable method for deep model learning. By putting a pre-trained model with some generalization ability onto a new similar dataset, the model will show superior performance than training a model from scratch after a simple and short training process. Similarly, the pre-trained model is adjusted for a specific dataset by a loss calculation function called model fine-tuning, and the loss calculation function widely used in deep neural networks today is the CrossentropyLoss (CE) (Shown in **Figure 2B**), which is calculated as follows:


(1) $$\text{Loss }=L\left(y,\hat{p}\right)=-y\log\;(\hat{p})-\left(1-y\right)\log\;(1-\hat{p})$$

where $\hat{p}$ is the predicted probability and y is the actual outcome category. A remarkable feature of this loss calculation is that the losses are also treated consistently for simple and easily scored samples ( $p_{t}\gg 0.5$ ). This result may also occur when the losses of a large number of simple samples accumulate and the small loss may swamp the sparse classes, or when there are differences in the loss of different classes in a near-saturated training process. There are two intuitive ways to deal with this type of problem, one is to add weighting factors directly to the loss function, and the other is to add adjustable factors represented by focal loss. In this paper, focal loss is introduced into the model by reducing the loss function to the weights of the easy samples, which can help the loss function to favour the difficult samples and improve the accuracy of the difficult samples (Shown in **Figure 2C**). The calculation procedure is as follows:


(2) $$Loss_{fl}=-{\left(1-p_{t}\right)}^{\gamma }\log\;\left(p_{t}\right)$$

where $p_{t}$ reflects the proximity to the ground truth. Larger $p_{t}$ means closer to category y, representing more accurate recognition. γ is the adjustable factor.

Due to the wide recognition of the ImageNet dataset, as well as the excellent performance and superior model fitting ability of the pre-trained models, all the experiments cited in this paper are based on the pre-trained models of the ImageNet dataset loaded with the corresponding models, effectively speeding up the training process of the models by means of transfer learning.

The FOTCA model studied in this article is mainly composed of a shortcut module and a Transformer module. The proposed overall model structure is shown in the **Figure 2A**, which is mainly divided into three steps: Patch and Position Embedding, Transformer architecture based on adaptive Fourier Neural Operators and Classifier.

#### 3.2.1 Patch and position embedding

The module consists of two sub-modules: Patch Embedding and Positional encoding based on learnable Fourier features. Compared with the regular position embedding, a primary advantage of Positional Embedding based on learnable Fourier features is that it provides richer positional information, especially for input from large datasets and long sequences. It can more finely encode each position through learnable Fourier basis functions, and this learnability makes it more flexible and adaptable. Additionally, it has better local connectivity and translational invariance when performing convolution operations, which can further enhance the model’s performance.

For the input image $X\in {\mathbb{R}}^{H\times W\times C}$ , Assuming we use a patch size of P×P , we can divide the input image into $\left(\frac{W}{P}\right)\times \left(\frac{H}{P}\right)$ local regions $P_{i,j}\in {\mathbb{R}}^{p\times pmesc}$ according to the patch size. ( $i\in \left[1,\frac{H}{P}\right],\;j\in \left[1,\frac{W}{P}\right]$ ). And expand it into a one-dimensional vector $v_{i,j}$ , whose size is D, where D is an adjustable parameter representing the vector dimension after patch embedding. For each matrix, we use a learnable weight matrix $W_{watch}$ of size D×K to map it to a low dimensional space:


(3) $$x_{i,j}^{\text{patch }}=W_{\text{patch}}\cdot v_{i,j}$$

There, $x_{i,j}^{\text{patch }}\in {\mathbb{R}}^{D\times K}$ is the feature vector obtained through patch embedding.

Next, we generate position encoding vectors for each patch. For each coordinate binary (i, j), use a learnable Fourier feature function to generate a vector $f_{i,j}^{pos}$ with a size of 2 K :


(4) $$\begin{array}{l} f_{i,j}^{pos}\left(p\right)=\left[\cos\;(\langle p,w_{1}\rangle +b_{1}\right),\sin\;(\langle p,w_{1}\rangle +b_{1}),\ldots ,\\ \cos\;(\langle p,w_{K}\rangle +b_{K}),\sin\;(\langle p,w_{K}\rangle +b_{K})] \end{array}$$

Where $p=\left(\frac{\left(i-1\right)}{H},\frac{\left(j-1\right)}{W}\right)$ is the normalized representation of input coordinates, $w_{K}$ and $b_{K}$ are learnable parameters, and 〈·,·〉 represents inner product between vectors. Finally, we add the position encoding vector to the feature vector obtained from patch embedding, resulting in the final feature vector $x_{i,j}^{pos}$ :


(5) $$x_{i,j}^{pos}=x_{i,j}^{patch}+f_{i,j}^{pos}$$

Then, we can use encoders to encode this sequence for further processing and recognition tasks.

#### 3.2.2 Adaptive Fourier Neural Operators

This module maps the input image to the frequency domain and uses adaptive Fourier basis functions to map each small block in the time domain to the frequency domain. This allows better extraction of frequency domain features and offers advantages in processing periodic as well as regular images, while providing better robustness to transformations such as rotation and scaling of the input data. Specifically, for each small block x , its frequency-domain representation ${\text{x}}^{\text{freq}}$ is obtained by first mapping it from the time domain to the frequency domain through Fast Fourier Transform (FFT). The ${\left(i,j\right)}^{th}$ element represents the frequency-domain representation at position (i,j) on the frequency domain.


(6) $${\text{X}}_{i,j}^{\text{freq}}=\sum_{m=0}^{P-1}\sum_{n=0}^{P-1}X_{m,n}e^{-2\pi i\left(\frac{mi}{P}+\frac{nj}{P}\right)}$$

Next, we will map each patch from the time domain to the frequency domain using adaptive Fourier basis functions. Specifically, for each small block ${\text{X}}_{i,j}$ , we calculate the inner product between it and the adaptive Fourier basis functions, and use the inner product value as coefficients.


(7) $$\alpha_{i,j,k}=\frac{1}{p^{2}}\sum_{u=1}^{p}\sum_{v=1}^{p}{\text{X}}_{i,j}\left(u,v\right)B_{u,v,k}$$

The coefficient of small block ${\text{X}}_{i,j}$ under the $k^{th}$ adaptive Fourier basis function is $\alpha_{i,j,k}$ , p is the size of the small block, and $B_{u,v,k}$ is the value of the $k^{th}$ adaptive Fourier basis function at position (u,v) . The specific calculation formula is as follows.


(8) $$B_{u,v,k}=\cos\;\left(\omega_{k}^{T}v_{u,v}\right)+i\cdot \sin\;\left(\omega_{k}^{T}v_{u,v}\right)$$

Where $v_{u,v}$ denotes the spatial location vector of the center point of the ${\left(u,v\right)}^{th}$ patch in the input image and $\omega_{k}$ represents the adaptive basis vector.

Expand all small adaptive Fourier basis functions into a large adaptive Fourier basis function matrix $\begin{array}{c} B\in {\mathbb{C}}^{p^{2}\times K} \end{array}$ , where K is the number of adaptive Fourier basis functions. At the same time, expand all small adaptive Fourier coefficients into a large adaptive Fourier coefficient matrix $\text{A}\in {\mathbb{C}}^{N\times K}$ , where N is the number of small blocks. Then, by performing global average pooling on A, obtain the weight $w_{k}$ of each basis function, and the calculation formula is:


(9) $$w_{k}=\frac{1}{\left(H/P\right)\times \left(W/P\right)}\sum_{i=1}^{H/P}\sum_{j=1}^{W/P}\alpha_{i,j,k}$$

The original data is ultimately mapped to the frequency domain through basis functions and basis function weights.


(10) $${\text{X}}_{i,j}=\sum_{k=1}^{K}w_{k}\cdot {\text{B}}_{i,j,k,:}\cdot {\text{x}}_{i,j,:}$$

Where $X_{i,j,:}$ denotes the flattened vector of patch (i,j). Finally, we merge the frequency-domain results of all patches into a large frequency-domain tensor $\text{F}\in {\mathbb{C}}^{\frac{H}{P}\times \frac{W}{P}\times k}$ . For channel mixing, it is done through an MLP on this frequency-domain tensor to extract high-level features.


(11) $$Z_{i,j}^{\left(k\right)}=\text{MLP }\left(F_{i,j}^{\left(k\right)}\right),i\in \left[1,\frac{H}{P}\right],j\in \left[1,\frac{W}{P}\right],k\in \left[1,N\right]$$

Finally, we concatenate all frequency domain features z together to form the final feature vector.


(12) $$Z=\left[z^{\left(1\right)},z^{\left(2\right)},\ldots ,z^{\left(F\right)}\right]$$

The feature vector can be fed into any type of neural network for further training or inference operations. This process has strong expressiveness and interpretability in data processing.

#### 3.2.3 Classifier

When using the Transformer-based model, its output vector is often passed to a classifier for image recognition. The traditional approach is to use the hidden layers of MLP to collect features and perform classification. However, there are many localized feature points in plant leaf disease images, and these localized feature points can usually be extracted and represented more efficiently by means of convolutional operations. Therefore, the use of CNNs may be more appropriate for such problems. Here, we consider using a basic block as a classifier, which includes convolutional layers and batch normalization layers, as well as shortcut operations. By upsampling to a higher dimension to obtain local feature values, feature transfer and information fusion are achieved. Also, shortcut operations can avoid gradient disappearance and model overfitting problems caused by excessive stacking of convolutional layers.

After downsampling or upsampling the feature maps using convolutional layers, the downsampled or upsampled feature maps are added to the output feature map using element-wise addition, which implements shortcut. This operation allows the model to better learn details and local features in the input, thereby improving the model’s performance. In this article’s classifier design, a 1×1 convolutional layer is embedded in the shortcut to adjust the depth of the feature maps so they can be added to the feature maps from the next layer. This helps preserve lower-level features, enhance inter-channel communication, and improve the model’s performance. Specifically, the input vector undergoes two 3×3 convolutional layers consecutively, with a certain amount of non-linear activation function (such as ReLU) inserted between them, as shown below:


(13) $${\text{x}}_{1}=\text{ReLU}\left(\text{Con}{\text{v}}_{3\times 3}\left(\text{x}\right)\right)$$

(14) $${\text{x}}_{2}=\text{ReLU}\left(\text{Con}{\text{v}}_{3\times 3}\left({\text{x}}_{1}\right)\right)$$

At the same time, downsampling is applied to the input vector to promote the underlying features towards the final recognition result. This operation can be achieved by using a 1×1 convolution layer in the shortcut. Finally, add the output of twoconsecutive convolution layers to the output of the shortcut, as shown below:


(15) $$\begin{array}{ll} {\text{x}}_{out} & =\text{ReLU }({\text{x}}_{2}+{\text{x}}_{shortcut})\\ =\text{ReLU }({\text{x}}_{2}+\text{ReLU }\left({\text{Conv}}_{1\times 1}(\text{x})\right)) \end{array}$$

Among them, ${\text{x}}_{shortcut}$ is the output of the shortcut, and it needs to ensure that its depth is the same as the output depth of the second convolution layer.

In the Classifier of this article, it has been demonstrated that shortcuts effectively alleviate the gradient vanishing problem, resulting in a decent convergence rate even when applied to very deep models. Using two 3×3 convolutional layers instead of a larger kernel increases nonlinearity, allows for faster collection and extraction of local features, and reduces the number of parameters, making the model easier to train and generalize.

## 4 Experiments and discussion

### 4.1 Experimental environment

The models we studied was developed based on the open source of deep learning framework pytorch1.11.0 with the following experimental equipment: CPU is Intel(R) Xeon(R) Platinum 8255C, GPU is single card V100-SXM2-32GB, CUDA version is 11.3, and programming language is Python3.8 (ubuntu20.04).

### 4.2 Experimental parameters, evaluation indicators

Based on earlier work by scholars (Kumar et al., 2023a; Kumar et al., 2023b; Wei et al., 2023), we selected DenseNet-169, Inception-v3, VGG19, ResNet-50, ResNet-101, ViT as our comparative experimental models to verify the feasibility and reliability of the improved model plant leaf disease image recognition method in this paper. We all iteratively update the pre-trained model parameters based on each model to accelerate the model convergence during the training process.

The training process uniformly uses the stochastic gradient descent SGD optimization algorithm to optimize its model. The same learning rate adjustment strategy is used for each parameter in the model, and the learning rate is dynamically adjusted using LambdaLR, which is adjusted by adjusting the learning rate according to the number of learning rate updates to linearly decay on top of the original one. The loss calculation function of the FOTCA model uses focal loss, and the rest of the models in the comparison experiments use the cross-entropy loss function. Dropout regularization is also used in the training process. By randomly dropping some neuron connections temporarily during the training process, the purpose is to effectively avoid overfitting of the model during the training process. The generalization ability of the model is also enhanced. The specific hyperparameter settings for the experiments are shown in **Table 1**.

**Table 1:** Hyperparameter tuning for the model.

Initial learning rate		0.001
Epochs		100
Batch size		8
Image size(for all)		224*224
Image size(for Inception-v3)		299*299
Transformer-based models	Embedding dimension	768
	Patch size	16
	Head number	8
	Depth	12

This table presents the various hyperparameters and their selected values used of the proposed FOTCA model and compared models.

We choose accuracy, adjustment time for accuracy, loss, adjustment time for loss, F1-score, parameters and FLOPS. We define for the first time the adjustment time for accuracy (loss). Adjustment time is the number of iterations required during model training to bring the model performance from the initial performance to 95% difference from the final converged performance. This metric reflects the ability of the model in terms of speed of convergence as well as training efficiency. The concept of adjustment time helps to deeply evaluate and compare the speed of the model training process. A shorter adjustment time means that the model reaches its potential performance faster within a limited number of iterations. Thus, with this metric, researchers can more intuitively assess the performance gap between different models.

In addition, adjustment time complements other commonly used evaluation metrics (e.g., accuracy, loss, etc.) to provide researchers with a more comprehensive view of how a model performs during training. In the case of similar model performance, shorter adjustment times may be an advantage due to greater savings in computational resources and time.

### 4.3 Comparative experiments

Firstly, the performance of Vision Transformer (ViT) is typically affected by the size of the training dataset due to the fact that ViT is based on transformers methods, which typically require a large amount of data for effective training. This is because transformers models, including ViT, are variants of the self-attention mechanism, which allows the model to capture the global relationships of the input data, but this mechanism also requires a large amount of data to support.

For the specific minimum sample requirement, it may vary as it depends on several factors, including the size of the model (e.g., the number of layers of the model, the number of hidden units, etc.), the complexity of the task (Lee et al., 2021) and the distribution of the data. Therefore, it can only be explored by trying the following experimental procedure.

**Figure 3:**
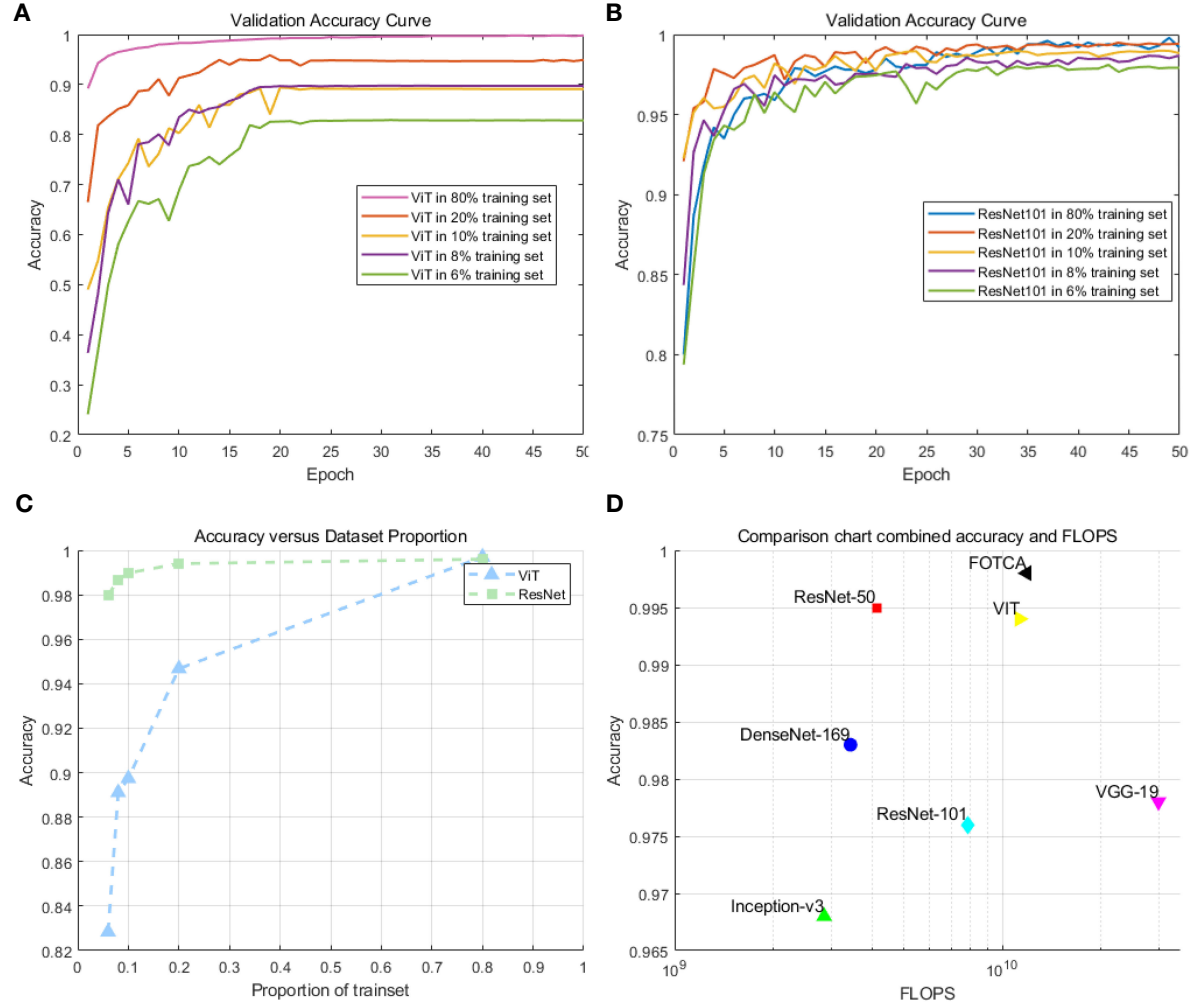
**(A)** Accuracy of the ViT model on small and medium-sized datasets: This plot demonstrates the training performance of the ViT model on datasets with different sizes. **(B)** Accuracy of the ResNet101 model on small and medium-sized datasets: This plotdemonstrates the training performance of the ResNet101 model on datasets with different sizes. **(C)** Comparison of the accuracy of ViT and ResNet101 for small and medium-sized datasets. **(D)** Scatter plot comparison of model accuracy and FLOPs(floating-point operations per second) for the models.

We chose to compare the ViT model with the ResNet101 model. This decision was made because ResNet101 has similar flops and comparable convergence capability to ViT on large-scale datasets. In preliminary experiments, we selected training set proportions of 6%, 8%, 10%, 20%, and 80%, respectively, to investigate the convergence capability and accuracy of ViT and ResNet101 on medium-sized datasets. The experimental results are shown in **Figures 3A–C**.

We observed that ViT’s training performance deteriorated rapidly on medium-sized datasets when the size of the training set was small. In contrast, ResNet101 demonstrated smaller variations and more stable curves, with minimal decreases in accuracy. Particularly, there was a significant turning point in ViT’s performance when the training set was at 8%. However, these experiments were not able to establish a definitive standard for determining the minimum sample requirements. On the contrary, the optimal dataset size may vary depending on specific applications and data characteristics. In many cases, if feasible, using larger datasets often yields better results.

**Figure 4:**
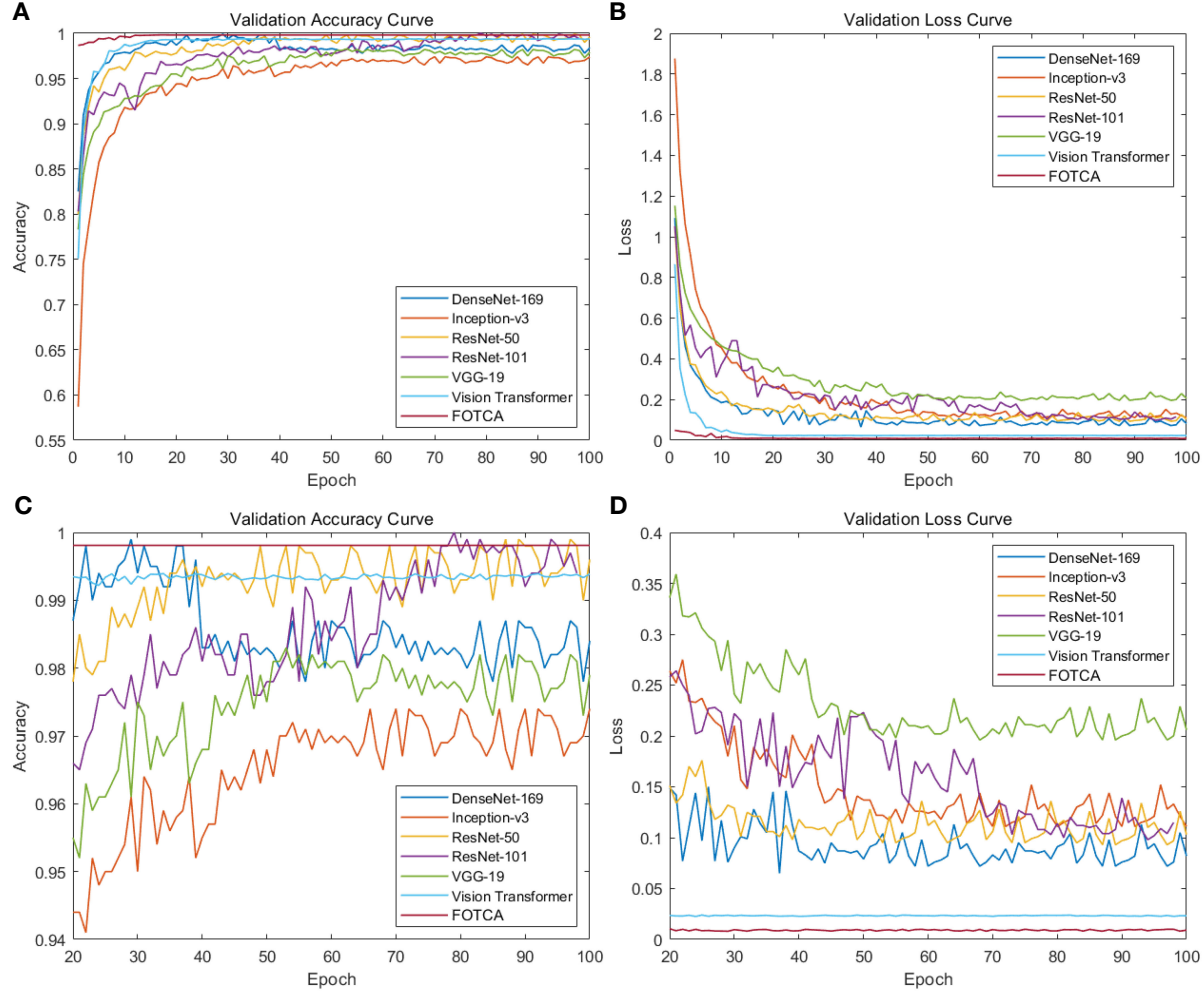
Showing training accuracy and loss comparison between the proposed FOTCA model and compared models in the study. **(A)** Training accuracy curve for all models. **(B)** Training loss curve for all models. **(C)** Partial training accuracy curve for all models. **(D)** Partial training loss curve for all models.

Next, we compared six mainstream image recognition models, using the same backbone training model and dataset to ensure the fairness of testing. The only exception is that the input image size must be 299×299 for the Inception-v3 model. We show the accuracy and loss values of each model during the iteration process in the chart. Due to the significant difference between the initial and final iteration results, the observation of the later iteration effect is not very clear and accurate. Therefore, we re-drew the chart showing the changes in the accuracy and loss values of later iterations between 20 and 100 iterations to achieve deeper analysis and optimization. This approach can enhance our understanding of the model’s performance, which helps us further improve and optimize our algorithms. The final experimental effect comparison is shown in **Figure 4**. Specifically shown in **Figure 3D**. The plot offers a visual representation of the balance between performance and computational efficiency among different models. The final result of the iteration is shown in **Table 2**.

**Table 2:** Performance metrics of the proposed FOTCA model and compared models.

Model	Accuracy(%)	Adjustment time	F1 score(%)	Loss	Adjustment time	Params*(M)*	FLOPS*(G)*
DenseNet169	98.3	13	91.85	0.082	14	14.15	3.44
Inceptionv3	96.8	28	94.45	0.15	29	23.83	2.86
ResNet50	99.5	21	98.23	0.103	27	25.56	4.13
ResNet101	97.6	32	95.69	0.107	32	44.55	7.87
VGG-19	97.8	28	91.42	0.206	30	78.14	29.96
ViT	99.4	9	98.03	0.026	9	58.07	11.28
FOTCA	99.8	11	99.31	0.005	9	59.14	11.87

Model Accuracy(%)Adjustment F1-Loss Adjustment Params(M) FLOPS(G).

According to the chart, the six mainstream image recognition models showed good feature extraction and representation abilities in the first $20^{th}$ epochs, with an accuracy of over 95% and a loss of below 0.35 for all of them. However, we observe a strange status quo demonstrated in the figure: the growth curve of the Transformer-based model is more stable, but the curve of the CNNs model fluctuates up and down consistently at 1% (in accuracy) and 0.03 (in loss), and DenseNet-169 even overfitted, with its accuracy and loss at the $29^{th}$ epoch values are the highest (99.8%) and lowest (0.072) of the whole iteration, respectively. Subsequently, the accuracy gradually declined and finally stabilized at about 98.3%. This phenomenon may be due to thedifferent learning patterns and model depths of CNNs and Transformer-based models(containing FOTCA and ViT) during the training process.The convolutional operation of CNN networks requires a large number of parameters. In contrast, the ViT network uses aself-attention layer instead of a fixed convolutional layer, which is better able to cope with long sequences of image features. The training error of ViT networks is usually less jittery due to the relatively simple training process of the self-attention layer. Meanwhile, the characteristics of the attention link are more suitable for training fine-grained plant leaf disease image recognition tasks, which is further demonstrated inside the section4.4. At the same time, overfitting is sooner or later as long as the network is large and deep enough, as indicated in (Hinton et al. (2012). The large depth of DenseNet-169 (model depth of 169) reflects the overfitting of the model more quickly.

Meanwhile, comparing the adjustment time of various models, ViT’s training results and FOTCA model performance are optimal, both achieving near-maximum effects at the 9*^th^* epoch. Other CNN models are relatively slower and require higher computational costs to achieve the same training effect. Therefore, FOTCA model exhibits efficiency and accuracy in feature extraction, learning ability, and model convergence efficiency, making it a more excellent image recognition model.

Two categories of performance can be identified in the comparison experiments. One is represented by models such as VGG-19, DenseNet-169 and Inception-v3, whose accuracy did not reach 98% but has already approached the best performance, so the trend is no longer rising. The other category is represented by models such as ViT, ResNet-50, and ResNet-101, which are based on FOTCA proposed in this article. During the iteration process, these models can approach an accuracy of 99.9%, which means that almost perfect image recognition results can be achieved through limited training. Additionally, FOTCA has leading positions in both accuracy and F1-score indicators compared to other models, and it is the only model with an F1-score of 0.9931. On the basis of efficient recognition using ViT, FOTCA further improves accuracy, loss, and F1-score without increasing parameter quantity and FLOPS compared to other models. This makes its performance nearly perfect, as shown in all the final parameters, parameter quantities, and FLOPS charts for all models in the experiment. Therefore, the FOTCA model proposed in this article achieves the best performance in plant leaf disease recognition.

### 4.4 Model visualization analysis

**Figure 5:**
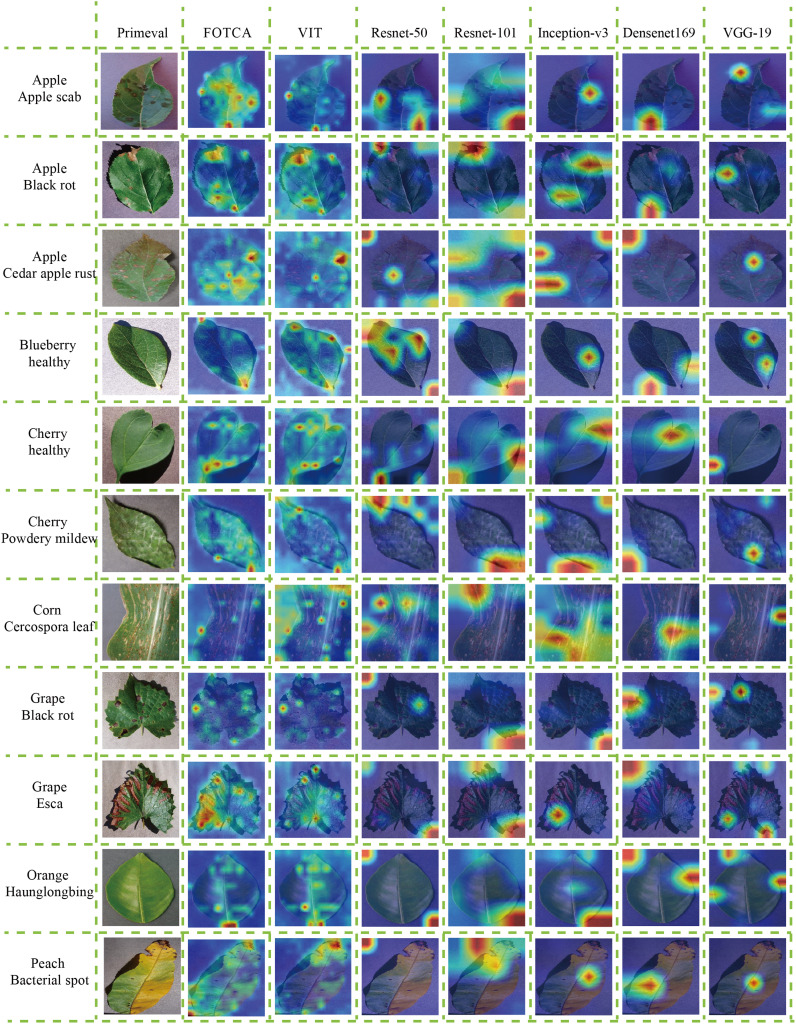
Attention heatmaps for the models on representative plant leaf disease images. The attention heatmaps visually demonstrate the regions within the image that the respective models focus on, highlighting their effectiveness at capturing relevant features for accurate recognition.

We proceeded to discuss the differences between the models in terms of their focus on image features by selecting 11 more representative photographs from the dataset that contained features that were essentially the basic features under the category. One photo of a healthy cherry leaf was taken with a global angle of view of the whole leaf and with a portion of the background included (this allows surveying the model’s ability to segment objects and backgrounds and verifying whether the model can focus more on the objects themselves), with the diseased and healthy traits shown on the leaf as curling at the edges of the diseased leaf and wilting of the whole leaf, and one partial image of a leaf with grey leaf spot A partial image of a maize leaf with grey leaf spot, which does not include the background (this verifies the model’s ability to focus on fine-grained features of the object and the size of the receptive field), and the trait of grey leaf spot on the maize showing random grey-brown rectangular long or irregular long spots and brown-black pockmarks throughout the leaf, with additional longitudinal correlation images having similar features. The graph shows the attention of the seven models to the same image, where the warmer the colour, the more attention the model pays to that feature, and the colour distribution gives an indication of the model’s ability to accurately identify the image features. Each model feature concern map is showing in the **Figure 5**.

First of all, both FOTCA and ViT models focus more accurately on the details of cherry leaf edges, but in the results, it was found that the ViT model focuses more on the whole leaf surface, which generates more errors and retains unnecessary information, thus reducing the recognition accuracy. At the same time, none of the CNNs can accurately and carefully focus on the correct plant leaf disease feature points, and the fluctuating anomalies of the metrics (including accuracy and loss values) during the training process are proved accordingly, and it also sideways proves the results that the training of Convolutional Networks is not as good as the ViT effect in the recognition of plant leaf disease images.

In addition, during the recognition of maize grey leaf spot leaves, the FOTCA and ViT models focused on the brown-black pockmarks on the leaves, and the ViT model focused more evenly on the background colour similar to the subject colour, Inception-v3 was able to observe the rectangular long stripes on the image more accurately, while the rest of the models focused on looser areas and could not reflect a more convincing pattern. In turn, the rest of the images show that the FOTCA model focuses more on the fine-grained features of the edges and less on the details of the whole leaf than the ViT model, and the five CNN models, on the other hand, have a more mixed focus, tending to focus on the global information and main features of the images. From this we can conclude that the Transformer-based models focuses more on the global features of the image, forming a global attention to the image, while the CNNs model focuses more on the subtle features, forming a local attention to the image.

### 4.5 Ablation experiment

**Figure 6:**
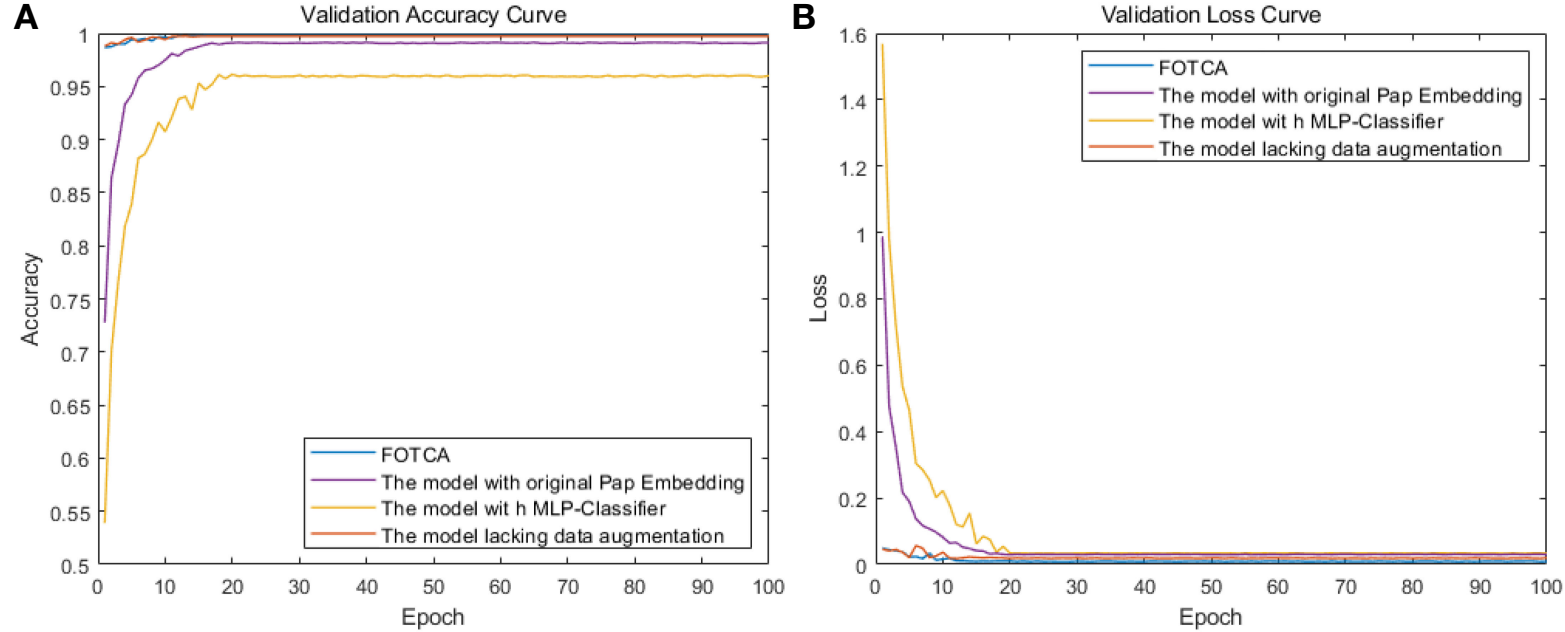
Showing training accuracy and loss comparison between the proposed FOTCA model and ablation experiment models with different parts removed. **(A)** Training accuracy curve for all models. **(B)** Training loss curve for all models.

To evaluate the effectiveness of each improvement module in the FOTCA model, we conducted ablation experiments using the original dataset. We compared four models: the FOTCA model, the FOTCA model with original Pap Embedding, the FOTCA model with MLP-Classifier, and the FOTCA model lacking data augmentation. We plotted the accuracy and loss curves for each model as a function of epoch value in **Figure 6**. Detailed training data is presented in **Table 3**. Partial iteration accuracy and loss values are not shown here because the comparison of the individual models in the results of this experiment can be shown more clearly in Figure.

**Table 3:** Performance metrics of the proposed FOTCA model and ablation study models with different parts removed.

Model	Accuracy(%)	Adjustment time	F1-score(%)	loss	Adjustment time
FOTCA	99.8	12	99.31	0.005	9
Using the original Pap Embedding	99.1	12	96.31	0.029	11
Using MLP-Classifier	96.1	12	85.91	0.0341	15
Missing data augmentation	99.7	11	98.94	0.019	12

#### 4.5.1 Between FOTCA model and the model using original pap embedding

Using Fourier transform can better capture texture and detail information in the image. This is because the Fourier transform can convert spatial-domain information into frequency-domain information, allowing the model to better process this information. In addition, using Fourier transform can greatly reduce the dimensionality of the vector and thus reduce computation complexity.

However, Fourier transformation may cause some spatial information loss, thereby affecting the performance of image recognition tasks. From the experimental results, it can be seen that using Pap Embedding with Fourier operator can partially improve the accuracy and convergence speed of the model, thus achieving better performance. Compared with using original Pap Embedding, the accuracy was improved by 0.7% and the loss decreased by 0.014.

#### 4.5.2 Between FOTCA model and the model using MLP − classifier

The classifier used in this article contains shortcut connections across layers, which can help information transmit more effectively, avoid gradient vanishing and information bottleneck problems, and has better explainability. In contrast, using MLP as the classifier will lose some important spatial structure information because MLP is a fully connected layer that flattens all features together and ignores their relationships. The classifier in this article can reconstruct spatial structure information of images more accurately, retain more feature information, and improve the model’s recognition accuracy. At the same time, as a classifier, it can help reduce the risk of overfitting by helping the model learn more robust features that have similar responses for different input data, thus improving the model’s generalization ability, which is also reflected in the training results.

#### 4.5.3 Between FOTCA model and the model lacking data augmentation

From the iteration graph, it can be observed that the model without data augmentation behaves extremely similarly to the original model during the process of iteration (the accuracy of the original model is 99.8%, and the accuracy of the comparative model is 99.7%). Even in the early stages of training, it produces better results than the original model, possibly due to the large amount of pest and disease data in this study, which allows for a sufficient sample size to collect fine-grained features without the need for data augmentation. However, continuous data augmentation during the initial stages of model training causes the model to learn orientation features while learning the original features of the photos, increasing the computational load of the model, resulting in a less effective performance on the test set compared to the model without data augmentation.

For the model using the original Pap Embedding, its learning ability is constrained for the same linkages, which adds limitations to the model architecture. As for the model using MLP-Classifier, its performance change is the largest among the four models, indicating significant contributions of the classifier used in this paper, which can significantly improve the fitting effect of the model. At the same time, the adjustment time of accuracy (loss) for the four experiments is roughly the same, indicating that making minor changes to the model architecture under the same model does not have a significant effect on convergence efficiency. The FOTCA model is still a fast-converging model that is leading in plant leaf disease image recognition.

## 5 Conclusions

Based on the above experimental results, the Transformer-based models outperforms the CNNs in the field of plant leaf disease image recognition, mainly because it allows a more detailed and accurate focus on image features., and can surpass the CNNs in terms of accuracy, loss, model matching speed, convergence efficiency, etc. The FOTCA model proposed in this paper can further improve its feature observation and extraction ability, and has a tendency to improve in accuracy, loss and F1-score, while demonstrating the enormous potential and application of adaptive Fourier operators. We will continue to extend our model in the future to combine diverse approaches for plant crop disease identification and detection in complex contexts in complex future contexts.

## Data availability statement

The original contributions presented in the study are included in the article/supplementary material. Further inquiries can be directed to the corresponding authors.

## Author contributions

BH contributed to the conceptualization and design of the study and constructed improved experiments. WJ and JZ organized data organization and analysis validation. CC and LH wrote some manuscripts. All authors contributed to manuscript revision, read, and approved the submitted version.
